# The Medical Problems of Flying

**Published:** 1917-12-15

**Authors:** 


### WAR IN THE AIR.

### The Medical Problems of Flying.

The creation of the Medical Research Committee is a good illustration of the truth that a wise plan sometimes produces good results outside the special area in which it was expected to operate. The Committee came into existence prior to the war and in connection with the administration of the National Insurance Act. As a side issue of this Act it was anticipated that much information would be gained relative to the incidence of disease, that this information would give an opportunity for a careful study of the causes of disease, and would thus lead to the enlargement of the sphere of preventive medicine. How far this anticipation has been justified or disappointed it is not now necessary to inquire. Imperative claims arising from the war were soon made on the Committee, and these claims have largely dominated its work during the last three years. All who know what has been undertaken and accomplished will agree that the existence of the Committee was a piece of good fortune, and that, had it not existed, many urgent situations which have been satisfactorily met would have been left in a sad state of defect. If in some directions the enterprises of the Committee have been interrupted, new opportunities, on the other hand, have been presented to it. Some of these have been urgent and peremptory, others have been, as it were, accidental. In all of them have been needed the knowledge and skill of highly-trained experts, special and often expensive apparatus, and an organisation to state the problems which required solution and to apply the solution to practical ends. The funds which have been drawn upon for these purposes have been, as already stated, provided by the scheme of the National Insurance Act, and they may fairly be described as an official endowment of scientific research. It may be hoped that the lesson will be taken to heart by all those who take a part in guiding the destinies of the nation. To institute a comparison between the money spent and the results accomplished would be to compare two considerations which have no common measure. All that can be said is that, judged on a severely economic basis, the expenditure has had a rich return, while the saving of human life, the restoration of human efficiency, and the establishment of confident knowledge constitute a chapter which is sure of an abiding record. Here, within the appreciation of every intelligent person, is a display of what is possible by the application of scientific method to the problems of daily life. If the particular illustrations are mainly afforded by the exigencies of war, the truths which are involved are not less true of the circumstances of peace, and the instrument which has served so well in the one set of conditions must be preserved and strengthened for service on more normal occasions.

With regard to the funds at the disposal of the Committee, a rather curious position has arisen. The original arrangement was that the annual amount available should be equal to the sum provided by the payment of a penny by each person insured under the National Insurance Act. Now, by the play of enlistment in the Army, the number of such persons has been largely diminished, whereas the anticipation was that by the natural expansion of the insured population the number would be increased. Consequently, the Committee finds itself with something like £7,000 per annum less than it had a right to expect. It will be strange indeed if from some source this defect is not remedied. The Treasury just now has to listen to many claims, good, bad, and indifferent. But it must give ear to this one, for none other can guarantee a more profitable return.

As an example of the special situations arising out of the war and demanding scientific response, we may mention the medical problems of flying. The rapidity of the movement, and the temperature and pressure conditions established by high altitudes, necessarily exercise a special influence upon the functions of the circulatory and respiratory mechanisms. Experience has shown that not in all cases can these mechanisms accommodate themselves to the new conditions. The individual may be quite sound as judged by ordinary medical and physiological standards, and may be quite effective in civil life, or even in military life as this is commonly understood. Yet he fails in the special circumstances created by the aeroplane. Obviously it became necessary to determine the tests by which men doomed to this failure could be picked out in advance. This has now been done, and a special body of medical practitioners is engaged on the practical application of these tests to recruits who offer themselves for the Air Service. The knowledge gained in this work is of high physiological interest, and in due time it will be placed at the service both of physiology and of practical medicine. So particular and so confident is it that its application enables an expert examiner to identify the man who can undertake the highest flights from one who must be restricted to lower levels.

Another practical issue important to the flying-man is the provision of oxygen in order to meet the inevitable deficiency of this gas in the rarefied air which the airman has to breathe. Obviously, questions of bulk and weight and convenience of apparatus are involved, and all of these have had to be faced. The mechanician, as well as the physiologist and the physician, has been necessary, and happily none has been wanting. The story of success is valuable in itself; but, in addition, it is extended, as is often the case with new truths, into unanticipated benefits. Thus from it have been learned an efficient and economical method of oxygen administration in respiratory diseases, as compared with the wasteful clinical fashion now practised; the value and use of the gas as a preventive of fatigue; and the possibility of improvements in the apparatus used in the depths of mines for the rescue of life.

Space forbids us to do more than select these few items from the work of the Research Committee. The full record will fill volumes, and will stand permanently as an announcement of the capacity of our scientific workers—of their industry, their ingenuity, and their genius. The nation generally has risen to the height of a great occasion, and has proved itself worthy of a great past. When the history of the time comes to be written the contribution made by science and medicine will be recognised as not the least effective of the factors which led to victory over a remorseless and ingenious enemy.

